# Sinus Augmentation—Expect the Unexpected: Diagnostic Anatomical Study

**DOI:** 10.3390/jcm10194293

**Published:** 2021-09-22

**Authors:** Bahaa Haj Yahya, Dror Bar-Hai, David Samehov, Gavriel Chaushu, Yafit Hamzani

**Affiliations:** 1 Oral and Maxillofacial Surgery Private Clinic, Herzliya 4672211, Israel; bahaa.hag@gmail.com; 2 Department of Oral and Maxillofacial Surgery, Beilinson Hospital, Rabin Medical Center, Petach Tikva 4941492, Israel; drorbh11@gmail.com (D.B.-H.); gabi.chaushu@gmail.com (G.C.); 3 Maccabi Dent Clinic, Bnei Brak 5144003, Israel; davidckoi@gmail.com; 4 Department of Oral and Maxillofacial Surgery, The Maurice and Gabriela Goldschleger School of Dental Medicine, Tel Aviv University, Tel Aviv 6139001, Israel

**Keywords:** big-nose variant, maxillary sinus, nasal cavity bone augmentation, dental implant

## Abstract

“Big-nose variant” is an anatomical phenomenon defined as the pneumatization of inferior third of the nasal cavity within the alveolar ridge while simultaneously displacing the maxillary sinus laterally. The purpose of the present study was to assess the prevalence of the big-nose variant phenomenon and suggest a morphology classification system. Diagnostic anatomical evaluation was performed in a tertiary medical center on 321 randomly selected maxillary cone beam computerized tomography scans of patients who presented at an oral and maxillofacial department. Two anatomical categories were defined for anatomical identification: classes for horizontal mesiodistal distribution, and divisions for vertical distribution. Class 2, defined as location of the nasal/sinus border between the distal edge of the canine up to the distal edge of second premolar, was found to be the most prevalent (64.6%). Class 3, defined as location of the nasal/sinus border distal to mesial edge of the first molar, was found in 17.9% of cases. Regarding the divisions category, in 96% and 58.2% of teeth examined, nasal cavity alone was found to be superior to the canine and first premolar, respectively, defined as Division A. In 46.9% and 85.6% of teeth examined, maxillary sinus alone was located above the second premolar and first molar, respectively, defined as Division C. Identifying Class 3 on the paraxial reconstruction is the first step in identifying big-nose variant, with further assurance gained from each determining division. The use of the classes and divisions may enable better maxillary treatment planning, alert surgeons for the unexpected, and avoid complications.

## 1. Introduction

The maxillary sinus and nasal cavity are two major anatomical units which dental surgeons face during implant placement in the posterior maxilla [1]. Typically, the maxillary sinus is stretched mesiodistaly (MD) between the first premolar and the second molar positions [2,3]. In the vertical dimension, the sinus floor is typically positioned 4–5 mm lower than the nasal floor [4,5]. Inferior nasal meatus pneumatization increases the extent of the nasal cavity toward the posterior direction, resulting in the displacement of maxillary sinus laterally and the nasal cavity inferiorly, in adjacent to the maxillary posterior teeth. Misch et al. [6] described this inferior turbinate and meatus pneumatization, as “big-nose variant”. This anatomical variant was found in 3% of 550 computed tomography (CT) scans of complete or partially edentulous maxillae [6]. A rhinology retrospective anatomical imaging study of 205 spiral, multi-planar, high-resolution, maxillary CT scans found big-nose variant’s prevalence at 4.88%, with a slight predisposition of females and a bilateral appearance [7].

“Big-Nose Variant” may not be noticed by dental surgeons, as the detection is especially challenged by two-dimensional radiographic views such as panoramic radiograph [1]. Three-dimensional cone beam computerized tomography (CBCT) was found to be significantly more reliable in the detection of sinus pathology compared to panoramic radiograph [8]. Nowadays, CBCT is considered the modality of choice for preoperative evaluation in implant dentistry [8,9]. Maxillary CBCT scan can aid in anatomical assessments such as maxillary sinus floor location; presence of sinus septum; thickness of lateral maxillary sinus walls; location and diameter of alveolar antral arteries; relationship of Schneiderian membrane to roots of adjacent teeth; quality of subantral bones; and locations of the border between the nasal cavity and maxillary sinus [1,8,9,10].

In cases with apparently sufficient bone volume, dental surgeons unaware of the “big-nose variant” phenomenon might place dental implants into the nasal cavity and inappropriately penetrate the inferior turbinate [1]. Facing inadequate bone volume of atrophic maxilla, intention for sinus bone augmentation might result in nasal floor augmentation, or inversely sinus floor augmentation can lead to creation of bone volume in a too distal maxillary area not intended for implant placement [6]. Hence, the surgeon should identify this anatomical variant preoperatively through CBCT then opt a proper treatment plan to ensure favorable surgical outcomes.

The aim of the present study was to assess the prevalence of this anatomical variant through CBCT scans and suggest a simple and easy-to-use morphology classification system for this phenomenon.

## 2. Materials and Methods

This retrospective research was conducted in the Oral and Maxillofacial Surgery Department of a tertiary medical center over one year (June 2018 to June 2019). The study sample consisted of 642 maxillary CBCT scans according to the following Inclusion criteria:

1. Available maxillary CBCT scans including cross-sections of 1–2 mm each;
2. Patients attended the oral and maxillofacial department due to their willing for maxillary dental implants placement;
3. Age >18 years old;
4. No history of chemotherapy or radiotherapy to the jaws;
5. No history of trauma or former surgeries to the jaws.

Of the 642 scans that met these criteria, 2 groups of 321 scans each were generated by a “block” randomization method (each block was composed of 3 scans). One group, which consisted of 321 scans, was chosen for review in the study. Hemi maxilla served as the statistical unit, rendering a total of 642 items.

Two evaluators (oral and maxillofacial resident and dentist) assessed each CBCT scan twice, and identified: (1) the horizontal MD border between the nasal cavity and maxillary sinus and (2) the vertical position of the nasal cavity in reference to the maxillary sinus and residual alveolar ridge. Inter-examiner reproducibility was evaluated using intraclass correlation coefficients, resulting in 0.92 (*p* < 0.05). A third evaluator (oral and maxillofacial surgeon) verified the data by random re-analysis of 66 scans. The medical center’s Helsinki Committee approved the study protocol (approval number 0396-16-RMC; 19 August 2020).

Data were collected from paraxial reconstructions (cross sections) extended from the maxillary canine to the first molar. Two spatial categories were defined, namely classes and divisions.

**Figure 1:**
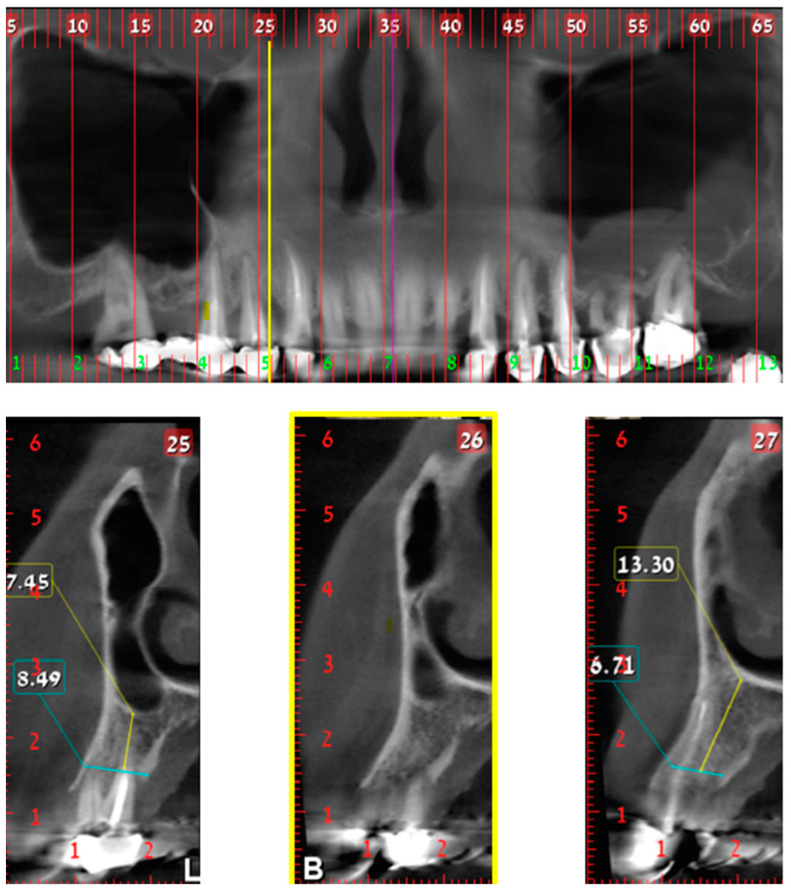
Class 1; Upper part—CBCT panoramic reconstruction; Lower part—from right to left—CBCT cross sections: cross section number 27—demonstrate nasal cavity alone above the alveolar ridge; cross section number 26—yellow line and frame—demonstrate nasal and maxillary sinus “border cross section”, which is located above the distal part of the canine, defined as Class 1; cross section number 25—demonstrate one cross section distal to the “border cross section”. B, buccal; L, lingual.

**Figure 2:**
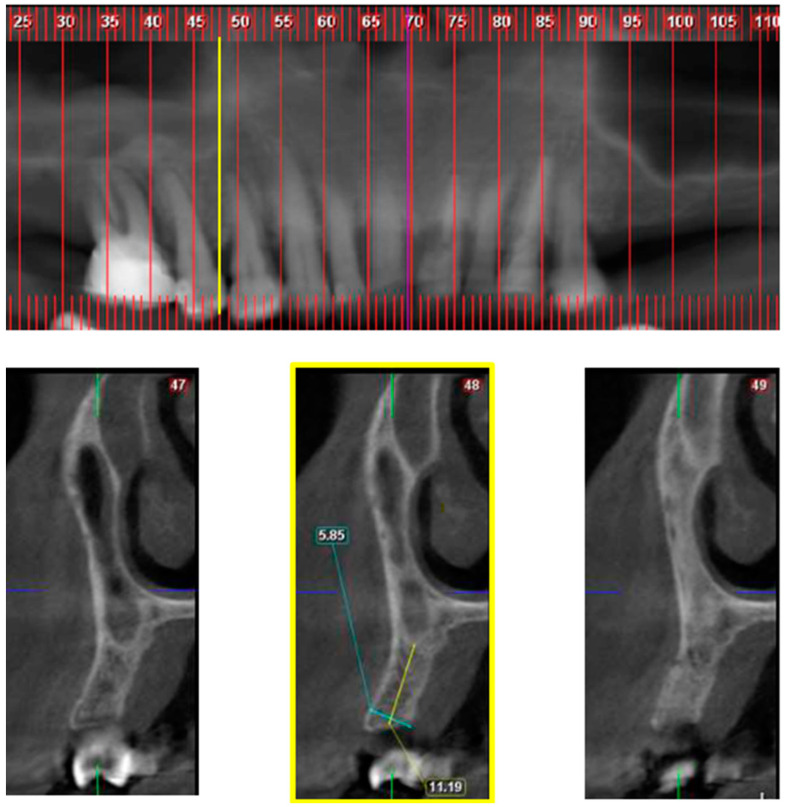
Class 2; Upper part—CBCT panoramic reconstruction; Lower part—from right to left—CBCT cross sections: cross section number 49—demonstrate nasal cavity alone above the alveolar ridge; cross section number 48—yellow line and frame—demonstrate nasal and maxillary sinus “border cross section”, which is located between 1st and 2nd premolars, defined as Class 2; cross section number 47—demonstrate one cross section distal to the “border cross section”.

**Figure 3:**
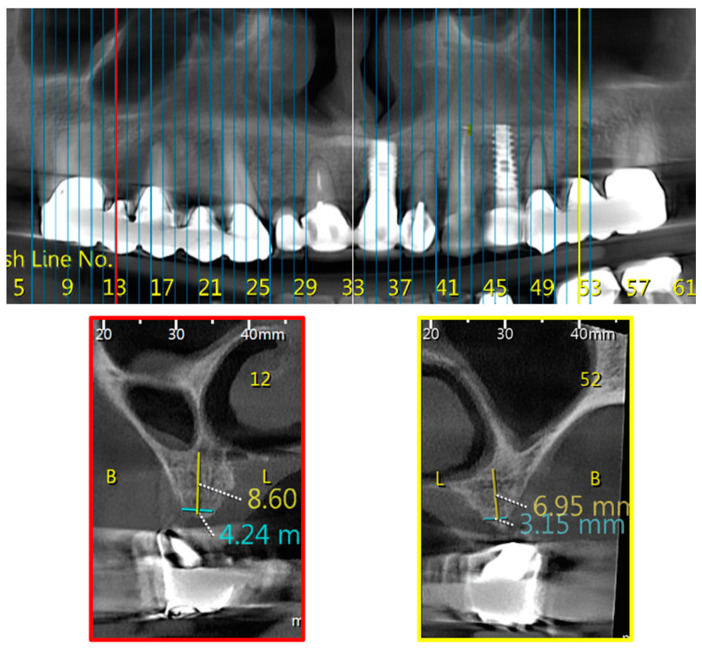
Bilateral Class 3; Upper part—CBCT panoramic reconstruction; Lower part—from right to left—CBCT cross sections: cross section number 52—demonstrate left nasal and maxillary sinus “border cross section”, which is located distal to the mesial edge of the 1st molar, defined as Class 3; cross section number 12—demonstrate right nasal and maxillary sinus “border cross section”, which is located distal to the mesial edge of the 1st molar, defined as Class 3. B, buccal; L, lingual.

Classes define the MD location of the nasal/sinus border for each hemi maxilla as demonstrate in Figure 1, Figure 2 and Figure 3. The “border cross section” is the most mesial cross section, in reference to maxillary midline, that above the alveolar crest has both nasal cavity and maxillary sinus cavity. The definition of classes is as follows:

Class 1—“border cross section” is located MD up to the distal edge of the canine.

Class 2—“border cross section” is located MD up to the distal edge of 2nd premolar.

Class 3—“border cross section” is located distal to the mesial edge of the 1st molar.

Classes 1 and 2 were defined as normal anatomy and Class 3 as “big-nose variant”.

**Figure 4:**
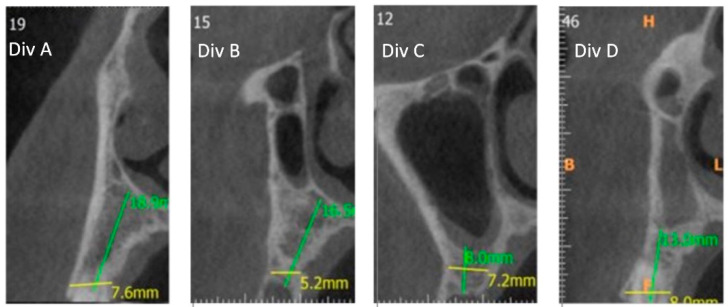
From left to right—CBCT cross sections: Division A (Div. A): Nasal cavity alone; Division B (Div. B): Nasal cavity in adjacent to sinus cavity; Division C (Div. C): Sinus cavity alone above the alveolar ridge; Division D (Div. D): Nasal cavity is positioned superiorly to sinus cavity.

Divisions define, for each tooth (i.e., canine to first molar), the vertical relationship between nasal and/or sinus cavity above the residual alveolar ridge as demonstrated in Figure 4.

Data regarding age and gender were collected from the patients’ medical files in order to evaluate their influence on the outcome parameters. The manuscript was written in compliance with the STROBE checklist.

Statistical analysis was conducted using SAS software, version 9.4 (SAS Institute Inc., Cary, NC, USA). Continuous variables are presented by mean and standard deviation, and categorical variables by number and percent. Student *t*-test was used to compare continuous variables between study groups. For categorical variables, we used Fisher exact test (for two values) or Chi-square test (for more than two values). Two-sided *p* values < 0.05 were considered statistically significant.

## 3. Results

The study group included maxillary CBCT scans of 321 patients (166 females, 155 males). Mean age was 48.9 ± 12.7 (range 19–92) years, and median age was 49 years.

### 3.1. Classes

MD distribution was as follows for all scans examined: Class 1—17.5%, Class 2—64.6%, and Class 3—17.9% (Table 1).

In the current study, 17.2%, 66%, and 16.8% of CBCTs examined were categorized as Class 1, 2, and 3, respectively, among 166 females; 18.6%, 62.4%; 19% were categorized as Class 1, 2, and 3, respectively, among 155 males. No gender related distribution differences were noted.

Evaluating Classes of both sides of maxillary CBCTs scans by age revealed Classes 1, 2, and 3 to be 18.8%, 63%, and 18.2%, respectively, among patients <50 years, with similar percentages (Class 1 = 18.8%, Class 2 = 65.4%, Class 3 = 15.8%) among older patients (>50 years). The two groups (above and below 50) were divided according to the mean and median age, no age-related distribution differences were noted.

### 3.2. Divisions

#### 3.2.1. Canine

The operating surgeon usually expects the nasal cavity to be superior to the canine. This anatomical situation coincides with Div. A occurring in 4% of cases (as Divisions B, C, and D require a different approach).

#### 3.2.2. 1st Premolar

The operating surgeon usually expects either the nasal cavity (58.2%) or the maxillary sinus (12.1%) to be superior to the 1st premolar. This anatomical situation coincides with Div. A and Div. C, respectively; Div. B and Div. D require a different approach, occurring in 29.7% of cases.

#### 3.2.3. 2nd Premolar

The operating surgeon usually expects either the maxillary sinus (46.9%) or nasal cavity (18.5%) to be superior to the second premolar. This anatomical situation coincides with Div. A and Div. C; Div. B and Div. D require a different approach, occurring in 34.6% of cases.

#### 3.2.4. 1st Molar

The operating surgeon usually (85.6%) expects the maxillary sinus to be superior to the 1st molar. This anatomical situation coincides with Div. C. Divisions A, B, and D requires a different approach, occurring in 14.4% of cases.

As noted, classes will be able to diagnose correctly only Div. A and Div. C, as diagnosis of Div. B and Div. D require further division analysis.

Greatest attention to the unexpected should be given to the second premolar, followed by the first premolar and first molar. The canine area remains the most predictable, although special attention should be given in 4% of cases.

Outcome parameters comparison regarding age (*p* = 0.53, Fisher’s Exact test), gender (*p* = 0.49, Fisher’s Exact test), and side (*p* = 0.8, Chi-Square test) did not show any statistically significant predisposition.

## 4. Discussion

Dental implant placement should be prosthodontic-guided, allowing for optimal restoration. The dental surgeon must ensure sufficient bone quantity and quality for the desired implant position. Atrophic maxilla treatments options can be divided into bone grafting procedures or non-grafting alternatives [10,11]. Among the former, sinus augmentation and nasal floor elevation are known surgical methods that enable the dental surgeon to achieve sufficient vertical bone height [1,12,13].

Scientific and clinical knowledge, combined with accurate CBCT analyses, aid dental surgeons in developing patient-specific treatment options [1]. Classes category, in the suggested classification, allow primary definition of the patient sinus/nasal border as normal (Classes 1, 2) or big-nose variant (Class 3), and assist in designing optimal maxillary dental implants treatment plans. Class 2 was found in the majority of cases (64.6%), pointing at the second premolar as the most frequent sinus/nasal border. Class 3 was found in 17.9% of the cases. Such distributions are more frequent than previously reported [6,7], emphasizing the need to be aware of big-nose variant during treatment planning.

Dental implant insertion within the inferior third of nasal cavity may cause various clinical manifestations such as nasal mucosa penetration, bleeding, inflammation, rhinosinusitis, and decrease in implant survival and success rates [1,14,15,16,17,18]. A recent study of 132 implants placing MD between the first premolar and second molar, showed 26 accidental penetrations to nasal cavity instead of sinus cavity during nasal endoscopy examination. According to endoscopy findings of six of these patients, the protruded parts of the implants were covered by non-inflamed nasal mucosa [1]. Moreover, the study presented a high survival rate (92.3%) of the implants that penetrated the nasal cavity, in contrast to an earlier study demonstrating normal nasal mucoperiosteum covering of the penetrated implants, with lower (72% over 5 years and 70% over 10 years) survival rates [14]. Other studies found that implant apical penetration magnitudes of more than 3 mm perforated the nasal mucosa [16,17,18] and as a result may cause infection [15,17]. Available data regarding the clinical manifestations are diverse, and thus dental surgeons should be aware and alert to avoid unplanned penetration of the nasal cavity.

Evaluating the relationship between the nasal cavity and the maxillary sinus on a tooth-by-tooth basis, one can conclude that in most cases (85.6%), sinus augmentation was needed when facing insufficient vertical bone for implant placement in the first molar position. Before placing a dental implant in canine position, dental surgeons should consider that in the majority (96%) of cases nasal floor is placed above. Therefore, if superior vertical bone augmentation will be needed, nasal floor elevation should be performed.

Inversely from the canine and first molar, placing dental implants in maxillary premolars position can surprise the dental surgeon facing a different cavity than expected. Although unexpected, in 18.5% of cases the nasal cavity is placed superior to second premolar, and in 12.1% of cases the maxillary sinus is located superior to the first premolar. Moreover, nasal and adjacent sinus cavity (Div. B) was found in 22.3% of first premolars and 30.8% of second premolars, requiring special consideration. First and second premolars are the most unexpected teeth, and thus CBCT evaluation at those positions should be conducted carefully in order to plan patient-specific dental implants.

Mismatching between prevalence of the present study’s big-nose variant (Class 3 = 17.9%) and Misch’s original prevalence (3%) [6] can be explained by different definitions of this anatomical variant. While the first definition is more general [6], the present classification defines the second premolars as the most frequent teeth to mark the border between the nasal and sinus cavities. Moreover, when sub classifying the second premolar to divisions, Div. D (Figure 4) counts for 3.8% (Table 2), and may be the anatomical variant Misch et al. [6] first defined, once nasal cavity displaced the maxillary sinus laterally and superiorly.

The major limitations of the study were basing the evaluation on complete or partially edentulous maxillae CBCTs without consideration of the teeth extraction effect on the sinus and nasal cavity pneumatization. Moreover, we did not take into account factors such as ethnicity, climate, smoking habit, and periodontal status of the patients. Future studies may evaluate the influence of those different factors on class and division distributions of big-nose variant [19,20,21].

## 5. Conclusions

The present study presents a modified interpretation of Misch’s definition of “big-nose variant” [6] and reveals its prevalence in the Israeli population. The proposed comprehensive classification tool may help the informed dental surgeon avoid complications, analyze clinical cases properly, and offer patients an improved maxillary dental implants plan.

## Figures and Tables

**Table 1:** Class distribution.

Class	Right Side N (%)	Left Side N (%)	Total N (%)
Class 1	52 (16.7)	54 (18.5)	106 (17.5)
Class 2	204 (65.4)	186 (63.7)	390 (64.6)
Class 3	56 (17.9)	52 (17.8)	106 (17.9)
Total	312 (100)	292 (100)	604 (100)

**Table 2:** Division distribution.

Division	Teeth N (%)				
	Canine	First Premolar	Second Premolar	First Molar	Total
Div. A	581 (96)	352 (58.2)	111 (18.5)	6 (1)	1050
Div. B	13 (2.2)	135 (22.3)	186 (30.8)	80 (13.2)	414
Div. C	2 (0.3)	73 (12.1)	283 (46.9)	518 (85.6)	876
Div. D	9 (1.5)	45 (7.4)	23 (3.8)	1 (0.2)	78
Total	605 (100)	605 (100)	603 (100)	605 (100)	2418

## Data Availability

Data supporting the reported results can be found in references [1,6,7] of the current manuscript.
